# A Renal Function Based Trade-Off Analysis of Non-vitamin K Antagonist Oral Anticoagulants in Nonvalvular Atrial Fibrillation

**DOI:** 10.3389/fphys.2018.01644

**Published:** 2018-11-20

**Authors:** Ling-Yun Zhou, Shuo-Fei Yang, Zhen Zhang, Chi Zhang, Long Shen, Zhi-Chun Gu, Xiao-Cong Zuo

**Affiliations:** ^1^Department of Pharmacy, The Third Xiangya Hospital, Central South University, Changsha, China; ^2^Department of Vascular Surgery, Renji Hospital, School of Medicine, Shanghai Jiaotong University, Shanghai, China; ^3^Pharmacy Department, Memorial Healthcare System, Hollywood, FL, United States; ^4^Department of Pharmacy, Renji Hospital, School of Medicine, Shanghai Jiaotong University, Shanghai, China; ^5^Department of Cardiology, Renji Hospital, School of Medicine, Shanghai Jiaotong University, Shanghai, China

**Keywords:** renal function, nonvalvular atrial fibrillation, non-vitamin K antagonist oral anticoagulants, trade-off analysis, dabigatran, rivaroxaban, apixaban, edoxaban

## Abstract

**Background:** Non-vitamin K antagonist oral anticoagulants (NOACs) depend on some degree of renal excretion, and no head-to-head comparisons based on renal function is available. This study mainly investigated the trade-off property of NOACs in nonvalvular atrial fibrillation (NVAF) with varying degrees of renal function.

**Methods:** A comprehensive search of Medline, Embase, Cochrane Library, and Clinical Trials.gov Website was performed for eligible randomized controlled trials (RCTs) that reported the efficacy and safety outcomes according to renal function of NOACs. Primary efficacy outcome was any Stroke or systemic embolism (S/SE). Major bleeding was considered as a primary safety outcome. Risk ratios (RRs) with their confidence intervals (CIs), the surface under the cumulative ranking curve (SUCRA), and trade-off analysis were conducted by renal function.

**Results:** Finally, 5 phase III Clinical Trials (72961 NVAF patients) comparing NOACs with warfarin in NVAF patients were included. In terms of normal renal function, dabigatran-150 mg was ranked first for efficacy (SUCRA: 90.3), and edoxaban-30 mg was ranked first for safety (SUCRA: 93.3). Dabigatran-110 mg/150 mg, and apixaban-5 mg were regarded as the most effective and reasonably safe interventions in the trade-off analysis. Regarding mild renal impairment, edoxaban-60 mg was ranked first for efficacy (SUCRA: 97.8), and edoxaban-30 mg was ranked first for safety (SUCRA: 99.5). Edoxaban-60 mg and dabigatran-150 mg were accounted as the most effective and reasonably safe interventions. With regards to moderate renal impairment, dabigatran-150 mg was ranked first for efficacy (SUCRA: 95.1), and edoxaban-15 mg was ranked first for safety (SUCRA: 98.2). Apixaban-2.5 mg and Edoxaban-30 mg was considered as the reasonably effective and the safest interventions.

**Conclusions:** Dabigatran-150 mg seems the most effective therapy in patients with normal renal function and moderate renal impairment, and edoxaban-60 mg in patients with mild renal impairment. Low dose edoxaban (15 and 30 mg) seems the safest intervention. Apixaban-2.5 mg and edoxaban-30 mg might be the best trade-off property in moderate renal insufficiency.

**HIGHLIGHTS**

Dabigatran-150 mg seems the most effective therapy for normal renal function and moderate renal impairment patients, edoxaban-60 mg for mild renal impairment patients.

Low-dose edoxaban can be considered as a good choice in NVAF patients at high risk of bleeding.

Apixaban-2.5 mg and edoxaban-30 mg might be the balanced option in NVAF patients with moderate renal insufficiency.

**STUDY REGISTRATION:**

PROSPERO Identifier, CRD42017054235.

## Introduction

Nonvalvular atrial fibrillation (NVAF) is the most common cardiac arrhythmia. In comparison to general population, AF is increasingly prevalent as kidney function declines (Alonso et al., 2011). Previous studies have revealed that AF and renal dysfunction share several common risk factors, such as hypertension, diabetes mellitus, and congestive heart failure (Olesen et al., 2012). Notably, both AF and renal dysfunction are associated with an increased risk of stroke and thromboembolic events (Olesen et al., 2012).

As Stroke/Systemic embolism (S/SE) is a leading cause of mortality and morbidity, it is important to identify individuals at potential high risk, and then appropriate therapy can be applied. Oral anticoagulation (OAC), whether with the vitamin K antagonists (VKAs, e.g., warfarin) or Non-vitamin K antagonist anticoagulants (NOACs), is an effective therapy to reduce the risk of S/SE in AF (Gu et al., 2018). Although, the efficacy of warfarin to reduce the incidence of S/SE in AF patients has been well-established in clinical trials for almost 60 years, the limitations of warfarin, such as the numerous food and drug interactions, have led to the development of NOACs (dabigatran, apixaban, rivaroxaban, and edoxaban) (Connolly et al., 2009; Granger et al., 2011; Patel et al., 2011; Hori et al., 2012; Giugliano et al., 2013). NOACs exhibit little potential for drug-drug or drug-food interactions, require no INR monitoring, and have been shown to be non-inferiority or superiority to warfarin in terms of stroke prevention and bleeding risk in phase 3 RCTs. Thus, based on their favorable benefit–risk ratio, international updated clinical guidelines on the management of AF have now issued a class I recommendation for the use of NOACs for stroke prevention in nonvalvular atrial fibrillation (NVAF) patients over warfarin (Kirchhof et al., 2016).

Whereas, it is important to realize that patients with advanced chronic kidney disease (CKD, creatinine clearance [CrCl] < 30 ml/min) and end-stage renal disease (ESRD) were excluded from all of the pivotal phase 3 NOAC trials (Connolly et al., 2009; Granger et al., 2011; Patel et al., 2011; Hori et al., 2012; Giugliano et al., 2013). Thus, no RCTs data guide the optimal management of coagulation for patients with AF and CKD. Notably, the 4 NOACs depend on some degree of renal excretion: dabigatran 80%, edoxaban 50%, rivaroxaban 36%, apixaban 27% (Chan et al., 2016). Consequently, NOACs may accumulate in patients with renal dysfunction, leading to an increased risk of bleeding. Despite elimination of warfarin is almost entirely by hepatic metabolism, warfarin could significantly increase the risk of bleeding as CrCl decreases secondary to the low time in therapeutic range and superimposed platelet dysfunction from worsen renal function (Chan et al., 2016). Additionally, patients with renal dysfunction are already at increased risk of bleeding (Gill et al., 2017). In general, the co-existence of AF and renal dysfunction results in a paradoxical increase in both thromboembolic and hemorrhagic risks. However, at this juncture, data and current clinical practice guidelines supporting the optimal strategy of OAC in NVAF patients with different stages of renal dysfunction remain limited. Previous studies only assessed the relative effect of the different OACs on thrombotic and bleeding risk in general AF patients (Tereshchenko et al., 2016; Sahay et al., 2017). In addition, there were some limitations of previous renal function-based analysis. Raccah et al. just assessed the safety of NOACs in NVAF or venous thromboembolism (VTE) patients with renal failure, and excluded from the analysis data of edoxaban 30/15 mg from the ENGAGE AF study (Raccah et al., 2016). Nielsen et al. did not extract enough information for the grouping of different dosage of apixaban or rivaroxaban, and did not provide the hierarchy of different treatments with respect to efficacy and safety (Nielsen et al., 2015). Although the latest renal function based analysis evaluated both efficacy and safety of OACs, all of the patients enrolled in the study had a moderate renal insufficiency (Andò and Capranzano, 2017). Thus, the clinical application is limited by their restricted generalizability. For an extensive picture on trade-off property with varying renal stratification, the aim of the present study is to summarize available evidences from NOAC RCTs, in order to carry out a rigorously pooled analysis, as well as, perform a decision-making on optimal OAC when regarding different renal function.

## Methods

### Data sources and search

The study was conducted in line with the standards of the Preferred Reporting Items for Systematic Reviews and Meta-Analyses (PRISMA) statement and the Cochrane Handbook. The protocol is documented online (PROSPERO registry: CRD42017054235). Databases of Medline, Embase, Cochrane Library were searched to identify all potential studies from inception to May 22, 2018. For the theme “NOACs,” we included the following terms: “dabigatran” or “Pradaxa” or “rivaroxaban” or “Xarelto” or “apixaban” or “Eliquis” or “edoxaban” or “Savaysa” or “betrixaban” or “Bevyxxa” or “Non-vitamin K antagonist oral anticoagulants” or “NOACs” or “direct oral anticoagulants” or “DOACs” or “novel oral anticoagulants” or “new oral anticoagulants” or “factor Xa inhibitors” or “factor II a inhibitors.” For the theme “atrial fibrillation,” the terms used were “atrial fibrillation” or “AF.” For the theme “RCTs,” we included the following terms: “randomized controlled trial” or “controlled clinical trial” or “clinical trial.” We used the Boolean operator “AND” to combine the three comprehensive search themes. In addition, unpublished trials were identified from the ClinicalTrials.gov Website. Two reviewers (Ling-Yun Zhou and Zhi-Chun Gu) independently examined the electronic searches and identified all potentially eligible studies. Disagreements were resolved by consulting a third author (Xiao-Cong Zuo).

### Study selection

We restricted our analysis to studies that were phase III RCTs and met all the following inclusion and exclusion criteria: (1) study consisting of randomized trial of patients with NVAF and receiving one of the NOACs as compared to warfarin; (2) study reporting the detail information about renal function and related outcomes of patients; (3) RCTs that included patients with prosthetic cardiac valves or mitral stenosis, mean or median follow-up < 6 months, < 200 participants, and NOAC phase II studies were not considered. For trials reporting more than one publication, data was extracted from the most complete publication, using the other reports to clarify or complement the information obtained. Two reviewers (Ling-Yun Zhou and Zhi-Chun Gu) independently assessed all study titles and abstracts for determining eligibility, and thereafter full paper was retrieved and assessed the relevant possibility according to the inclusion criteria. All discrepancies and uncertainties were resolved by consulting a third author (Xiao-Cong Zuo).

### Study outcomes

Primary efficacy outcome was any stroke (ischemic or hemorrhagic) or systemic embolism (S/SE). Major bleeding served as a primary safety outcome. Information about these outcomes was stratified by renal function according to CrCl using the Cockcroft-Gault formula, and classified in the following groups: CrCl>80mL/min (normal renal function), CrCl 50–80 mL/min (mild renal impairment), and CrCl < 50 mL/min (moderate renal impairment).

### Data extraction, quality evaluation, and bias assessment

The data extracted from each study included study identifiers (the name of study, year of publication); characteristics of individual study (number of patients, age, sex, type of atrial fibrillation); qualifying risk factors (Age>75 years, stroke, heart failure, hypertension, diabetes mellitus); CHADS_2_ score; time in therapeutic range of warfarin group; renal function; characteristics of drug intervention (NOAC dose, warfarin dose, co-medication with aspirin, duration of follow-up). Detailed data by renal function that was not reported in the original publications was further extracted from the US FDA databases (www.fda.gov). Quality of the study was analyzed using the Cochrane Collaboration Risk of Bias Tool, which include selection bias (method of randomization, allocation concealment), information bias (masking of outcome adjudicators), and bias in the analysis (intention to treat analysis, completeness of follow-up) (Wei et al., 2018). Potential publication bias was explored using visual inspection of funnel plots if the number of included studies was more than 10 (Wei et al., 2018).

### Statistical analysis

Both direct and indirect comparisons were performed using the STATA statistical software (version 13.0, Stata Corporation, College Station, TX, USA). The different treatment strategies were treated as separate nodes. Individual studies and pooled estimates were derived and presented in forest plots. Results were reported as relative risks (RRs) with their 95% CIs. The between-study heterogeneity was assessed through I^2^ test and Q statistic. I^2^ of >50% indicated considerable heterogeneity, and a *p*-value of < 0.05 at Q statistic represented a significant heterogeneity (Wei et al., 2018). For indirect comparison between the different NOACs using warfarin as the reference comparator, multivariate random-effect analysis was performed on a data set of point estimates. Inconsistency was appraised by comparing direct and indirect estimates. Ranking of evaluated interventions was performed to provide a hierarchy by employing the surface under the cumulative ranking curves (SUCRA). SUCRA is a relative ranking measure based on cumulative probability plots, which accounts both for the location and the variance of all relative treatment effects. The larger the SUCRA value, the better the rank of the treatment (Tereshchenko et al., 2016). For trade-off analysis, clustering methods were used to produce clusters of treatments to account for both efficacy and safety. Sensitivity analysis was conducted by excluding RCTs with inconsistent renal function hierarchy, with small sample, and with different population analyses (intention-to-treat population analyses or per-protocol population analyses) (Yan et al., 2018).

## Results

### Study selection and characteristics of included studies

**Figure 1:**
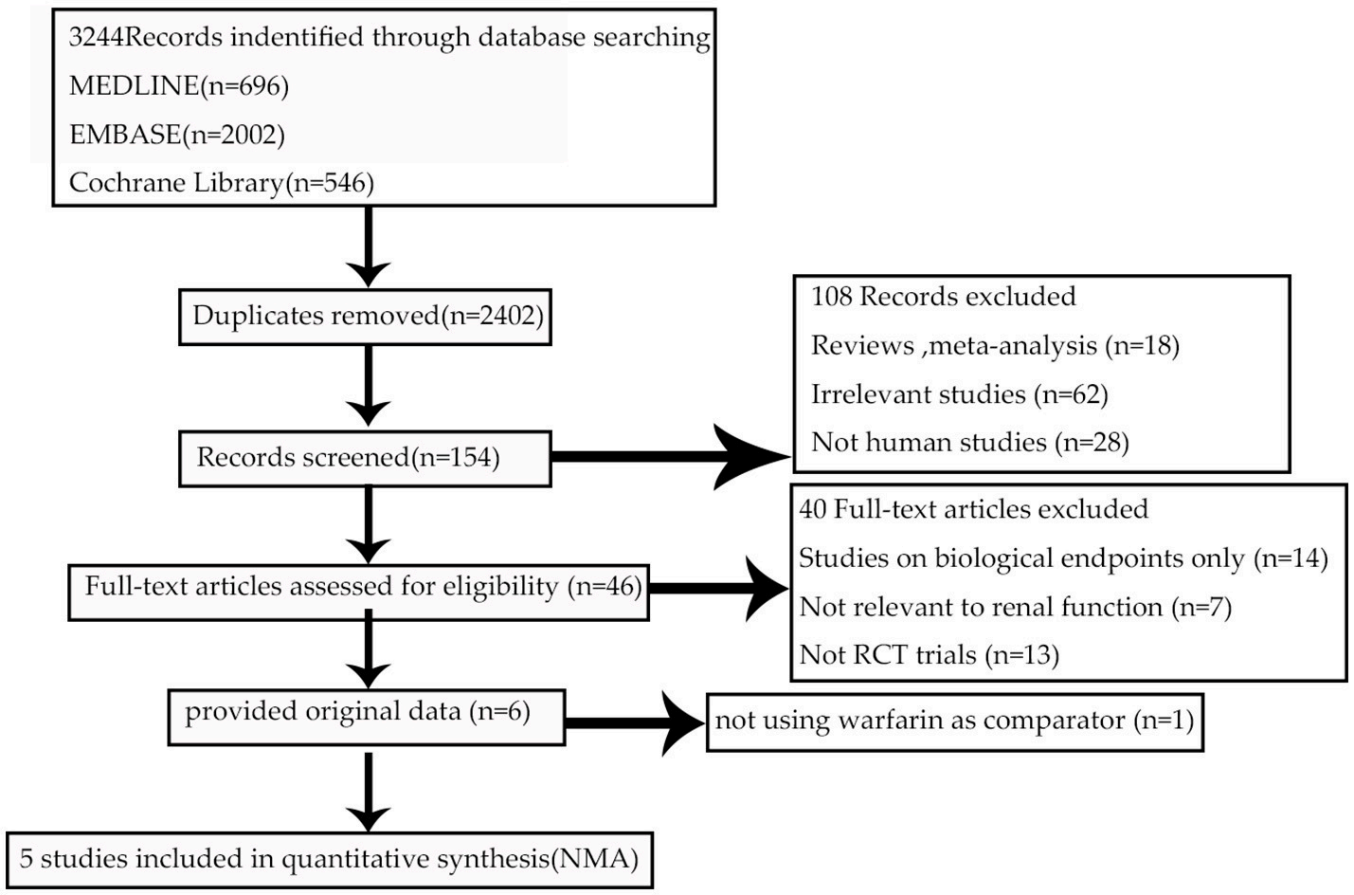
Flow diagram for the selection of eligible randomized controlled trials.

**Figure 2:**
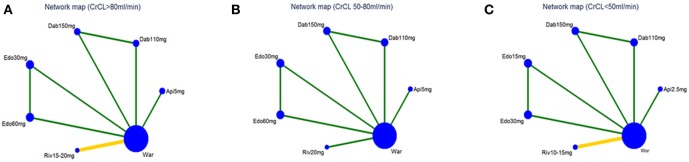
Network map for patients with **(A)** normal renal function, **(B)** mild renal impairment, and **(C)** moderate renal impairment. Nodes show interventions being compared. Edges represent direct comparison between pairs of interventions. The color of edges represents the level of bias in the majority of included studies in each comparison (green = low; yellow = unclear). War indicates Warfarin. Dab 110 mg indicates Dabigatran 110 mg. Dab 150 mg indicates Dabigatran 150 mg. Riv 10–15 mg indicates Rivaroxaban 10–15mg. Riv 15–20mg indicates Rivaroxaban 15–20mg. Api 2.5mg indicates Apixaban 2.5 mg. Api 5 mg indicates Apixaban 5 mg. Edo 15 mg indicates Edoxaban 15 mg. Edo 30 mg indicates Edoxaban 30 mg. Edo 60 mg indicates Edoxaban 60 mg.

The flow of references through the review was shown in Figure 1. Initially, our search strategy identified 3,244 articles with 842 duplicates. We reviewed the titles and abstracts of all articles and excluded 2,248 articles. After systematically reviewing the remaining 154 full texts, 46 articles fulfilled the inclusion criteria, but only 6 articles provided original data from RCTs. The AVERROES study was excluded from the analysis, as it utilized aspirin, not warfarin, as comparator. Finally, 5 RCTs comparing NOACs with warfarin in AF patients were included in the present analyses: RE-LY (Randomized Evaluation of Long-term Anticoagulation Therapy) trial, comparing dabigatran with warfarin; ARISTOTLE (Apixaban for Reduction in Stroke and Other Thromboembolic Events in Atrial Fibrillation) trial, comparing apixaban with warfarin; ROCKET AF (Rivaroxaban Once Daily Oral Direct Factor Xa Inhibition Compared with Vitamin K Antagonism for Prevention of Stroke and Embolism Trial in Atrial Fibrillation) trial and J-ROCKET AF, comparing rivaroxaban with warfarin; ENGAGE AF-TIMI 48 (Effective Anticoagulation with Factor Xa Next Generation in Atrial Fibrillation) trial, comparing edoxaban with warfarin (Connolly et al., 2009; Granger et al., 2011; Patel et al., 2011; Hori et al., 2012; Giugliano et al., 2013). 5 RCTs included 72961 NVAF patients with a median age of 71.5 years, and 36.9% were females. Median length of follow- up was 2.0 years. Clinical characteristics of the included RCT populations were reported in Table 1. J-ROCKET AF, ROCKET-AF, ARISTOTLE, and ENGAGE AF-TIMI 48 were double-blind and double-pacifier studies. RE-LY trial had the dabigatran arm blinded and the warfarin arm un-blinded. RE-LY and ARISTOTLE studies enrolled subjects at low risk of stroke (CHADS_2_ score of 2.1 both), whereas ENGAGE AF-TIMI 48 trial enrolled subjects with average CHADS_2_ scores of 2.8. J-ROCKET AF trial had the subjects with average CHADS_2_ scores of 3.3 for patients with rivaroxaban treatment and 3.2 for patients with warfarin treatment, and ROCKET-AF trial had the subjects at highest risk (CHADS _2_ score of 3.5). The time in therapeutic range (TTR) in the warfarin arms was the lowest in the ROCKET-AF trial (58%), similar in the ARISTOTLE, ENGAGE AF-TIMI48, and RE-LY trials (66% to 68%), and unclear in J-ROCKET AF trial. As shown in Table S1, the included studies satisfied all bias tool items except for RE-LY, which was not double-blinded. Figure 2 showed the renal function based network map, and warfarin is the common comparator across the studies. Inconsistency analysis did not conduct as all the comparisons resulting from indirect comparison.

**Table 1:** Summarized Characteristics of Included Trials.

	RE-LY (***n*** = 18113)			ROCKET-AF (***n*** = 14264)		J-ROCKET AF (***n*** = 1278)		ARISTOTLE (***n*** = 18201)		ENGAGE AF-TIMI 48 (***n*** = 21105)		
	**Dab 110 mg (*n* = 5957)**	**Dab 150 mg (*n* = 6029)**	**War (*n =* 5965)**	**Riv 15/20 mg (*n =* 7073)**	**War (*n =* 7081)**	**Riv 10/15 mg (*n =* 639)**	**War (*n =* 639)**	**Api 2.5/5 mg (*n =* 9080)**	**War (*n =* 9042)**	**Edo 15/30mg (*n =* 6919)**	**Edo 30/60mg (*n =* 6884)**	**War (*n =* 6922)**
**CHARACTERISTIC**												
Age (year)	71.4	71.5	71.6	73.0	73.0	71.0	71.2	70.0	70.0	72.0	72.0	72.0
Female (%)	35.7	36.8	36.7	39.7	39.7	17.1	21.8	35.5	35.0	38.8	37.9	37.5
**TYPE OF AF (%)**												
Paroxysmal AF	32.1	32.6	33.8	17.5	17.8	NR	NR	15.1	15.5	26.1	24.9	25.3
Persistent AF	67.8	67.4	66.1	81.1	80.8	NR	NR	84.9	84.4	73.5	74.7	74.3
**RISK FACTOR (%)**												
CHF	32.2	31.8	31.9	62.6	62.3	41.3	40.2	35.5	35.4	56.6	58.2	57.5
Hypertension	78.8	78.9	78.9	90.3	90.8	79.5	79.5	87.3	87.6	93.5	93.7	93.6
Age>75 year	NR	NR	NR	43.5	43.5	39.4	38.5	31.2	31.1	39.9	40.5	40.1
Diabetes mellitus	23.4	23.1	23.4	40.4	39.5	39.0	37.1	25.0	24.9	36.2	36.4	35.8
Stroke	19.9	20.3	19.8	54.9	54.6	63.8	63.4	19.2	19.7	28.5	28.1	28.3
MI	16.8	16.9	16.1	16.6	18.0	7.0	8.3	14.5	13.9	NR	NR	NR
Concomitant aspirin use	40.0	38.7	40.6	36.3	36.7	38.0	34.7	31.3	30.5	28.7	29.4	29.7
CHADS_2_score	2.1	2.1	2.1	3.5	3.5	3.3	3.2	2.1	2.1	2.8	2.8	2.8
0–1 (%)	32.6	32.2	30.9	0.0	0.0	0.0	0.0	34.0	34.0	0.0	0.0	0.0
2 (%)	34.7	35.2	37.0	13.0	13.1	15.2	18.0	35.8	35.8	46.0	46.0	47.0
3–6 (%)	32.7	32.6	32.1	87.0	86.9	84.8	82.0	30.2	30.2	54.0	54.0	53.0
**RENAL FUNCTION**												
CrCL < 50 ml/min	1196	1232	1126	1490	1459	141	143	1502	1515	1274	1287	1297
CrCL50-80 ml/min	2803	2852	2898	3298	3400	328	328	3817	3770	3034	2985	3030
CrCL>80 ml/min	1958	1945	1941	2285	2222	170	168	3761	3757	2611	2612	2595
TTR (median, IQR)	67 (54–78)			58 (43–71)		NR		66 (52–77)		68 (57–77)		
Follow up (years)	2.0			1.9		2.5		1.8		2.8		
Population analyses	ITT			ITT		PP		ITT		ITT		

*Dab, dabigatran; Riv, rivaroxaban; Api, apixaban, Edo, edoxaban; AF, atrial fibrillation; CHF, Congestive Heart failure; MI, Myocardial infarction; CrCL, Creatinine clearance; TTR, time in therapeutic range; IQR, interquartile range; ITT, intention-to-treat population analysis; PP, per-protocol population analysis*.

### Outcomes in patients with normal renal function (CrCl>80 ml/min)

**Figure 3:**
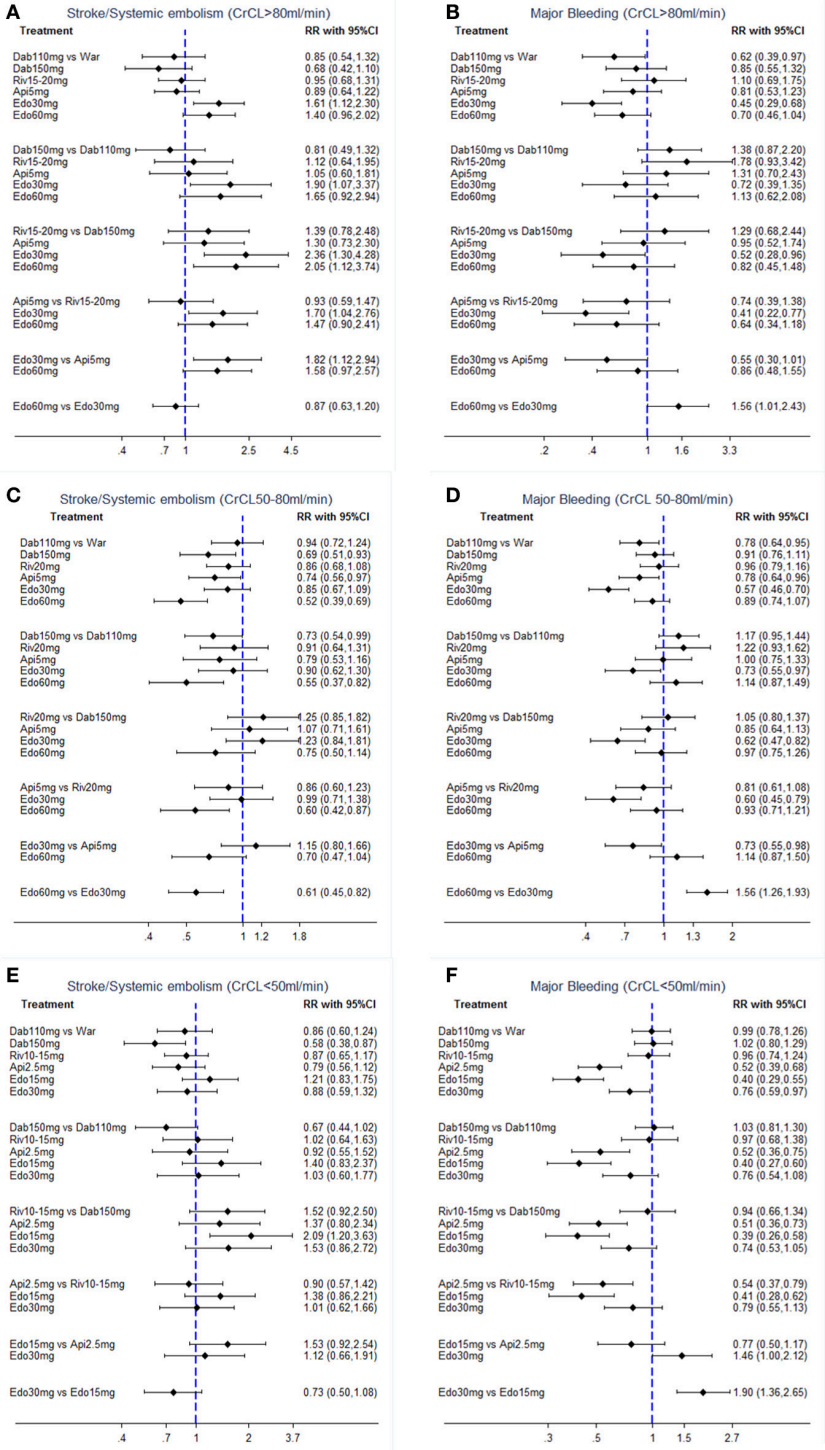
Forest plot for efficacy and safety in patients with **(A,B)** normal renal function, **(C,D)** mild renal impairment, and **(E,F)** moderate renal impairment. War indicates Warfarin. Dab 110 mg indicates Dabigatran 110 mg. Dab 150 mg indicates Dabigatran 150 mg. Riv 10–15 mg indicates Rivaroxaban 10–15mg. Riv 15–20 mg indicates Rivaroxaban 15–20 mg. Api 2.5 mg indicates Apixaban 2.5 mg. Api 5 mg indicates Apixaban 5 mg. Edo 15 mg indicates Edoxaban 15 mg. Edo 30 mg indicates Edoxaban 30 mg. Edo 60 mg indicates Edoxaban 60 mg.

In terms of efficacy, no NOAC was significantly better than warfarin. Furthermore, treatment with edoxaban-30 mg significantly increased the risk of S/SE as compared to all the other OACs (RR:1.61 95%CI: 1.12–2.30 as compared to warfarin, 2.36 [1.30–4.28] as compared to dabigatran-150 mg, 1.90 [1.07–3.37] as compared to dabigatran-110 mg, 1.70 [1.04–2.76] as compared to rivaroxaban-15/20 mg and 1.82 [1.12–2.94] as compared to apixaban-5 mg) except for edoxaban-60 mg (0.87 [0.63–1.20]). Dabigatran-150 mg was superior to edoxaban, either 30 mg (0.42 [0.23–0.77]) or to 60 mg (0.49 [0.27–0.89]), in the odds of S/SE (Figure 3A).

The comparative safety results were shown in Figure 3B. Compared with warfarin, only dabigatran-110 mg (0.62 [0.39–0.97]) and edoxaban-30 mg (0.45 [0.29–0.68]) were associated with a significant reduction in major bleeding. Among NOACs, significantly reduced rate of major bleeding was observed only with edoxaban-30 mg vs. dabigatran-150 mg (0.52 [0.28–0.96]) or rivaroxaban-15/20 mg (0.41 [0.22–0.77]). Edoxaban-60 mg showed a significantly increased risk of major bleeding compared with edoxaban-30 mg [1.56 [1.01–2.43]].

### Outcomes in patients with mild renal impairment (CrCl 50–80 ml/min)

In terms of efficacy, dabigatran-150 mg (0.69 [0.51–0.93]) and edoxaban-60 mg (0.52 [0.39–0.69]) significantly reduced the risk of S/SE by around 30–50% when compared with warfarin. Among NOACs, treatment with edoxaban-60 mg significantly reduced the risk of S/SE as compared to all other OACs [0.55 [0.37–0.82] as compared to dabigatran-110 mg, 0.60 [0.42–0.87] as compared to rivaroxaban-20 mg and 0.61 [0.45–0.82] as compared to edoxaban-30 mg] except for dabigatran-150 mg (0.75 [0.50–1.14]). Dabigatran-150 mg provided the significant reduction in S/SE compared to dabigatran-110 mg (0.73 [0.54–0.99]) (Figure 3C).

The comparative safety results were shown in Figure 3D. In comparison to warfarin, treatment with dabigatran-110 mg (0.78 [0.64–0.95]), apixaban-5 mg (0.78 [0.64–0.96]) and edoxaban-30 mg (0.57 [0.46–0.70]) was associated with a significantly reduced rate of major bleeding. Edoxaban-30 mg was the safest OACs, demonstrating the significantly lowest risk of major bleeding (0.57 [0.46–0.70]) as compared to warfarin, 0.73 [0.55–0.97] as compared to dabigatran-110 mg, 0.62 [0.47–0.82] as compared to dabigatran-150 mg, 0.60 [0.45–0.79] as compared to rivaroxaban-20 mg, 0.73 [0.55–0.98] as compared to apixaban-5 mg, and 0.64 [0.52–0.79] as compared to edoxaban-60 mg].

### Outcomes in patients with moderate renal impairment (CrCl < 50 ml/min)

The comparative efficacy results were shown in Figure 3E. In comparison to warfarin, only dabigatran-150 mg (0.58 [0.38–0.87]) was associated with a significant reduction on S/SE. Furthermore, there was no statistically significant difference among NOACs.

In terms of safety, in comparison to warfarin, treatment with apixaban-2.5 mg (0.52 [0.39–0.68]), edoxaban-15 mg (0.40 [0.29–0.55]), and edoxaban-30 mg (0.76 [0.59–0.97]) was associated with a significantly reduced rate of major bleeding. In comparisons between NOACs, the risk of major bleeding was lower for patients taking apixaban-2.5 mg or edoxaban-15 mg as compared to patients taking other NOACs (Figure 3F). Edoxaban-30 mg showed a significantly higher risk of major bleeding when compared to edoxaban-15 mg (1.90 [1.36–2.65]).

### Ranking of NOACs for efficacy and safety

Table 2 demonstrated the ranking of NOACs. In NVAF patients with normal renal function, dabigatran-150 mg was the winner for the efficacy (SUCRA: 90.3; probability: 66.3%), and edoxaban-30 mg was the safest intervention (SUCRA: 96.3; probability: 82.3%). In NVAF patients with mild renal impairment, edoxaban-60 mg was the most effective treatment (SUCRA: 97.8; probability: 88.7%), and edoxaban-30 mg was the safest intervention (SUCRA: 99.5; probability: 97.1%). In NVAF patients with moderate renal impairment, dabigatran-150 mg was the most effective treatment (SUCRA: 95.1; probability: 80.3%), and edoxaban-15 mg was the safest intervention (SUCRA: 98.2; probability: 89.2%).

**Table 2:** SUCRA Ranking of OACs for efficacy and safety stratified by renal function.

	**Efficacy (S/SE)**			**Safety (major bleeding)**		
**Treatments**	**SUCRA**	**Pr. Best**	**Mean Rank**	**SUCRA**	**Pr. Best**	**Mean Rank**
**CrCL>80 ML/MIN**						
Warfarin	47.5	0.3	4.2	18.4	0.0	5.9
Dabigatran 110 mg	68.4	13.8	2.9	74.2	14.1	2.5
Dabigatran 150 mg	90.3	66.3	1.6	38.9	0.5	4.7
Rivaroxaban 15–20 mg	56.6	6.8	3.6	14.1	0.1	6.2
Apixaban 5 mg	66.5	12.7	3.0	46.5	1.6	4.2
Edoxaban 30 mg	4.1	0.0	6.8	96.3	82.3	1.2
Edoxaban 60 mg	16.7	0.1	6.0	61.6	1.5	3.3
**CrCL 50 TO 80 ML/MIN**						
Warfarin	9.4	0.0	6.4	10.4	0.0	6.4
Dabigatran 110 mg	23.1	0.0	5.6	69.5	1.3	2.8
Dabigatran 150 mg	73.6	8.1	2.6	34.4	0.0	4.9
Rivaroxaban 20 mg	39.8	0.1	4.6	24.8	0.0	5.5
Apixaban 5 mg	64.3	3.0	3.1	69.2	1.6	2.8
Edoxaban 30 mg	42.1	0.0	4.5	99.5	97.1	1.0
Edoxaban 60 mg	97.8	88.7	1.1	42.3	0.0	4.5
**CrCL<50 ML/MIN**						
Warfarin	26.7	0.0	5.4	23.6	0.0	5.6
Dabigatran 110 mg	52.4	1.5	3.9	26.7	0.0	5.4
Dabigatran 150mg	95.1	80.3	1.3	21.4	0.0	5.7
Rivaroxaban 10–15 mg	51.3	2.5	3.9	32.0	0.0	5.1
Apixaban 2.5 mg	66.0	10.5	3.0	84.7	10.8	1.9
Edoxaban 15 mg	7.9	0.0	6.5	98.2	89.2	1.1
Edoxaban 30 mg	50.6	5.1	4.0	63.3	0.0	3.2

*SUCRA, the surface under the cumulative ranking curve; Pr. Best, probability of being the best; S/SE, stroke or systemic embolism*.

### Trade-off analysis

**Figure 4:**
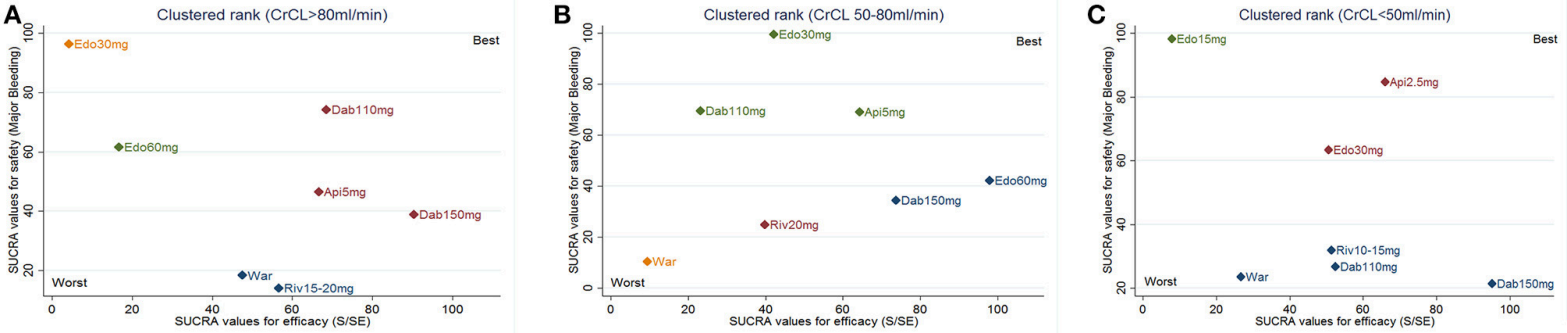
Trade-off analysis results based on renal function. The plot is based on cluster analysis of surface under the cumulative ranking curves (SUCRA) values. Each plot shows SUCRA values for two outcomes: primary efficacy (stroke or systemic embolism; S/SE) and safety (major bleeding). Each color represents a group of treatments that belong to the same cluster. Treatments lying in the upper right corner are more effective and safer than the other treatments. **(A)** for CrCl > 80 mL/min; **(B)** for CrCl 50–80 mL/min; **(C)** for CrCl < 50 mL/min. War indicates warfarin; Dab indicates dabigatran; Edo indicates edoxaban; Riv indicates rivaroxaban; Api indicates apixaban.

Trade-off analyses of NOACs were shown in Figure 4. The upper right corner in Figures 4A–C is empty, which means that a treatment balancing both efficacy and safety does not exist. In NVAF patients with normal renal function, clustered ranking revealed 4 separate clusters (Figure 4A). Dabigatran-110 mg, dabigatran-150 mg, and apixaban-5 mg formed a cluster of “the most effective and reasonably safe” interventions. Warfarin and rivaroxaban-15/20 mg formed a cluster of “the moderate effective and the most dangerous” interventions. Edoxaban-30 mg was the single representative of a cluster of “ineffective, but the safest.” Edoxaban-60 mg formed a separate cluster of “low effectiveness and moderate safety.” In NVAF patients with mild renal impairment, clustered ranking revealed 4 separate clusters (Figure 4B). Dabigatran-150 mg and edoxaban-60 mg formed a cluster of “the most effective and reasonably safe” treatment. Edoxaban-30 mg, dabigatran-110 mg, and apixaban-5 mg formed a cluster of “reasonably effective and the safest.” Rivaroxaban-20 mg represented “reasonably effective and reasonably safe” cluster. Warfarin formed a separate cluster of “low effective and the most dangerous.” In NVAF patients with moderate renal impairment, clustered ranking revealed 3 separate clusters (Figure 4C). Apixaban-2.5 mg and edoxaban-30 mg formed a cluster of “reasonably effective and reasonably safe.” Warfarin, dabigatran-110 mg, dabigatran-150 mg, and rivaroxaban-10/15 mg formed a cluster of “moderate effective and the most dangerous.” Edoxaban-15 mg was the single representative of a cluster of “low effective, but the safest.”

### Sensitivity analyses

The results of sensitivity analysis did not change by excluding RCTs with inconsistent renal function hierarchy, with small sample, and with different population analyses, confirming the robustness of the primacy findings (Figures S1–S10).

#### Publication bias

Funnel plot was not performed owing to the limited number of included studies (5 RCTS).

## Discussion

Renal dysfunction has been associated with increased risk of both thromboembolic and hemorrhagic events (Chan et al., 2016). The best choice of a compound among OACs for S/SE prevention in NVAF patients with different renal functions remains unclear. Based on the results of 5 RCTs, international updated clinical guidelines on the management of AF have issued a class I recommendation of NOACs for stroke prevention over warfarin in general NVAF patients (Kirchhof et al., 2016). However, no direct recommendation is stated about the choice of warfarin or NOACs for stroke prevention in patients with renal dysfunction. Furthermore, as a highly accumulated concentrations of NOACs in patients with impaired renal function, several reports have shown a high risk of bleeding when NOAC are prescribed for patients with renal insufficiency (Pengo et al., 2012). Therefore, it is difficult for clinicians to choose proper therapeutic intervention in NVAF patients with varying degrees of renal function. For this reason, we conducted a renal function based trade-off analysis of NOACs in 72,961 NVAF patients. The results indicated that NVAF patients with different stages of renal function should be matched to individual anticoagulation treatment for balancing both efficacy and safety.

The present study identified 5 RCTs comparing NOACs with warfarin. Similar with traditional analysis (Sardar et al., 2014), our results suggested that risk of S/SE would be significantly reduced in patients receiving individual NOAC when given a recommended dose, and the NOACs had a favorable safety profile compared with warfarin regardless of renal function. In addition, dabigatran-150 mg was superior to warfarin in decreasing the risk of S/SE in patients with mild and moderate renal impairment, only dabigatran-110 mg was associated with a lower rate of major bleeding in patients with normal renal function and mild renal impairment. Apixaban-5 mg reduced the risk of S/SE by 26% and the risk of major bleeding by 21% as compared to warfarin in patients with mild renal impairment. Apixaban-2.5 mg, edoxaban-15 mg, and edoxaban-30 mg had a similar efficacy to warfarin for protection against S/SE and had a safer profile than warfarin in patients with moderate renal impairment. Our results were consistent with previous studies, which showed that both doses of dabigatran, edoxaban or apixaban were no inferior to warfarin in decreasing the risk of S/SE without increasing the risk of major bleeding in general AF patients. As we known, good quality anticoagulation control (as reflected by average time in therapeutic range, TTR) is associated with better safety and efficacy outcomes on warfarin (Gallagher et al., 2011). And, although warfarin is primarily metabolized by the cytochrome P450 enzyme, dose requirement to maintain therapeutic anticoagulation for warfarin may be influenced by renal function (Limdi et al., 2010). Thus, the poor TTR and unadjusted dose of warfarin in different stages of renal function may be the reason that NOACs were favored compared to warfarin for reducing S/SE and major bleeding in present analysis.

The indirect comparison of our analysis confirmed the notion that there is a trade-off between efficacy and safety of 5 medications and that a single most effective and safest intervention does not exist in different stages of renal function. In patients with normal renal function, dabigatran-150 mg had the highest probability of being the most effective for prevention of S/SE, without increasing risk of major bleeding over other OACs except for edoxaban-30 mg. In patients with mild renal dysfunction, edoxaban-60 mg demonstrated probability (88.7%) of being ranked the most effective intervention, without increasing risk of major bleeding over other OACs except for edoxaban-30 mg. In patients with moderate renal impairment, dabigatran-150 mg showed the highest probability of being the most effective antithrombotic intervention, but, at the price of a greater risk of major bleeding, as compared to apixaban-2.5 mg and edoxaban-15 mg. Therefore, this trade-off analysis did not reveal obvious winners and confirmed the substantial overlap in the efficacy and safety of individual treatments in AF patients with different stages of renal function. Yet, it seems that the low-dose edoxaban (30 mg for normal renal function and mild renal impairment patients, 15 mg for moderate renal impairment patients) was favorable in all indirect comparisons for safety across the range of renal function. The safety profile of low-dose edoxaban was indeed evident in ENGAGE AF-TIMI 48 trial ancillary analysis and edoxaban-15 mg has been indicated for patients with creatinine clearance levels ranging from 15 to 30 mL/min in Japan (Bohula et al., 2016). For efficacy, dabigatran-150 mg was significantly favored compared to other NOACs in patients with normal renal function or moderate renal impairment. However, caution is warranted when dabigatran is used in patients with moderate renal impairment, given 80% renal excretion of this drug (Chan et al., 2016). For both efficacy and safety, in the present analysis, it appears that apixaban-2.5 mg was associated with the highest probabilities of being selected over other NOACs in patients with CrCL < 50 mL/min. The reason may be that apixaban, being the compound with the lowest renal clearance (26%), might be the NOAC with the best balance between benefits and risks in patients with moderate renal dysfunction (Chan et al., 2016). However, it should be taken into account that patients with renal dysfunction enrolled in ARISTOTLE trial were at lower risk, with lower CHADS_2_ scores and less frequent of heart failure, than those enrolled in the other 4 RCTs (Granger et al., 2011).

Therefore, by clinically interpreting the statistical results of this ranking NOACs analysis in the light of the aspects discussed above, it can be presumed that dabigatran-150 mg might be more likely considered as reasonable option for AF patients with normal renal function, edoxaban-60 mg for patients with mild renal impairment or apixaban-2.5 mg for patients with moderate renal impairment, respectively. Low-dose edoxaban can be considered as a good choice in cases with multiple risk factors of bleeding.

### Study limitations

Nevertheless, this analysis also had some limitations. Firstly, differences in patient populations, such as demography, stroke risk, and performance of warfarin control, and design of different trials, different cutoffs for defining renal impairment and different definitions of primary outcomes, remain unsolved issues in present analysis. In RE-LY trial, the randomization to dabigatran or warfarin was open-label but the dose of dabigatran was blinded. With the respect to patient population, an intention-to-treat (ITT) analysis was employed in ARISTOTLE and ROCKET- AF trials only for efficacy outcomes, not safety outcomes. In addition, the ENGAGE AF-TIMI 48 trial used a modified ITT, not a standard ITT, where patients were treated with edoxaban in a pre-defined study period. Dose adjustment methods were different in different trials. Rivaroxaban was dose-adjusted to 15 mg for patients with a CrCl of 30–49 mL/min, whilst apixaban was dose-adjusted to 2.5 mg for patients with≥2 of the following criteria: age≥80 years old, body weight ≤ 60 kg and creatinine level of 1.5 mg/dl. In the ENGAGE AF-TIMI 48 trial, the edoxaban dose was halved (in both intervention arms) at baseline and/or during follow-up if any of the following criteria were present: a creatinine clearance of 30–50 mL/min, body weight ≤ 60 kg or concomitant use of verapamil or quinidine (protocol amendment added use of dronedarone to this criterion). Notably, the used statistical techniques somewhat assume that the patients entered in the various trials and the level of anticoagulation were comparable. Secondly, common to most pooled analyses is the lack of individual patient data. Thus, we were compelled to select summary RR for analysis by measuring only the number of events and taking no account of when they occurred. Hence, the present results have to be interpreted with caution in light of the above-mentioned limitations. However, the clinical interpretation of indirect comparisons may guide practical decision-making in the absence of direct evidences about effects that most likely will not be investigated head to head in a randomized fashion.

## Conclusions

Overall, NOACs were significantly better than warfarin in terms of both safety and efficacy in patients with AF across all renal function. Dabigatran-150 mg seems the most effective therapy for normal renal function and moderate renal impairment patients, whereas edoxaban-60 mg might be the most effective intervention for mild renal impairment patients. Low-dose edoxaban can be considered as a good choice in cases with multiple risk factors of bleeding. Apixaban-2.5 mg and edoxaban-30 mg might be the best trade-off property for moderate renal insufficiency. The present study might be potentially guide physicians in selecting the most appropriate NOAC for each AF patient when regarding renal function.

## Author contributions

Z-CG and X-CZ are the guarantors of the entire manuscript. L-YZ, ZZ, and S-FY contributed to the study conception and design, data acquisition, analysis, and interpretation, drafting of the manuscript, critical revision of the manuscript for important intellectual content, and final approval of the version to be published. CZ and LS contributed to the data acquisition, analysis, and interpretation.

### Conflict of interest statement

The authors declare that the research was conducted in the absence of any commercial or financial relationships that could be construed as a potential conflict of interest.
