# Supplementary material for: A Renal Function Based Trade-Off Analysis of Non-vitamin K Antagonist Oral Anticoagulants in Nonvalvular Atrial Fibrillation
Source: Front Physiol. 2018 Nov 20;9:1644. doi: 10.3389/fphys.2018.01644 (PMC6256743; doi:10.3389/fphys.2018.01644)
Supplement: Supplementary file 1 [file Table_1.DOCX]

SUPPLEMENTAL MATERIAL

**Table S1. Quality assessment results of included Randomized Controlled Trials**

| **Study** | **Random sequence generation** | **Allocation concealment** | **Blinding of participants and personnel** | **Blinding of outcome assessment** | **Incomplete outcome data** | **Selective reporting** | **Other bias** | **Summary risk** |
| --- | --- | --- | --- | --- | --- | --- | --- | --- |
| RE-LY [1] | L | L | H | L | L | L | L | L |
| ROCKET-AF [2] | L | L | L | L | L | U | L | L |
| J-ROCKET AF [3] | U | U | U | U | L | L | L | U |
| ARISTOTLE[4] | L | L | L | L | L | U | L | L |
| ENGAGE AF-TIMI 48 [5] | L | L | L | L | L | L | L | L |

L: low risk; U: unclear risk; H: high risk.

**Figure S1: Sensitivity Analysis of Forrest Plot for Efficacy in Patients with Normal Renal Function**


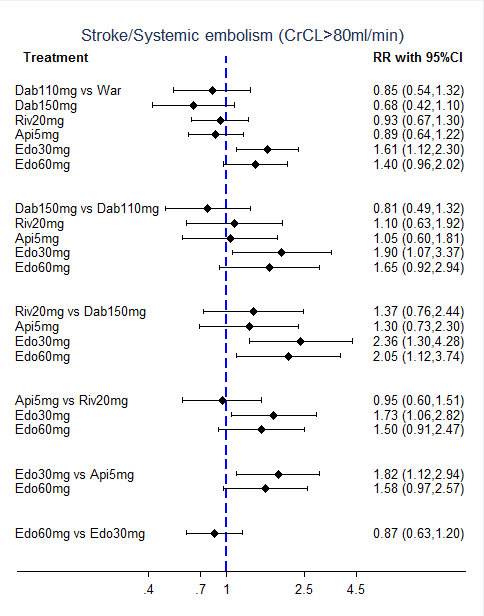


**Figure S2: Sensitivity Analysis of SUCRA Ranking for Efficacy in Patients with Normal Renal Function**


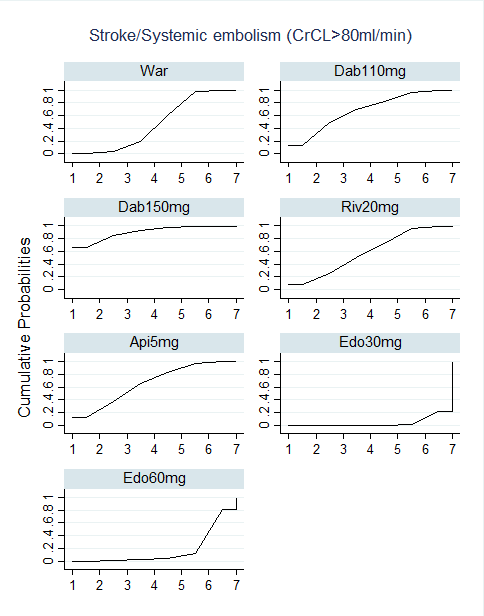


**Figure S3: Sensitivity Analysis of Forrest plot for Safety in Patients with Normal Renal Function**


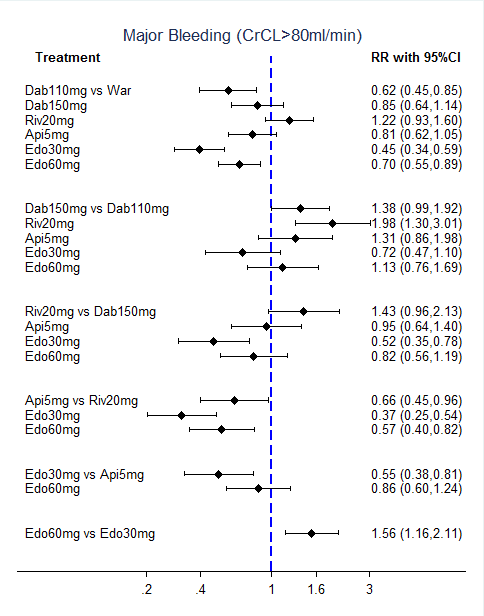


**Figure S4: Sensitivity Analysis of SUCRA Ranking for Safety in Patients with Normal Renal Function**


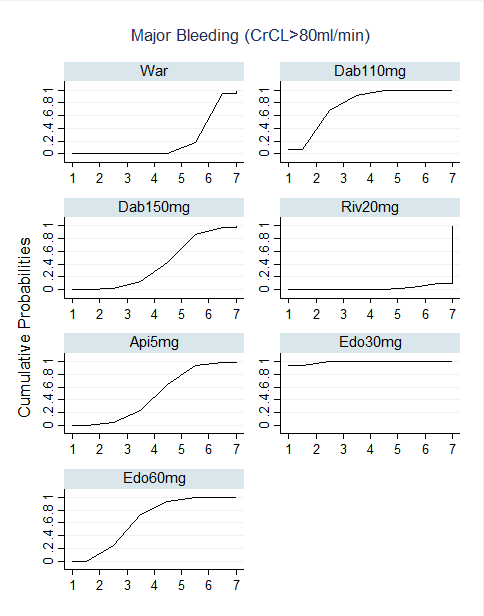


**Figure S5: Sensitivity Analysis of Clustered Ranking for Both Efficacy and Safety in Patients with Normal Renal Function**


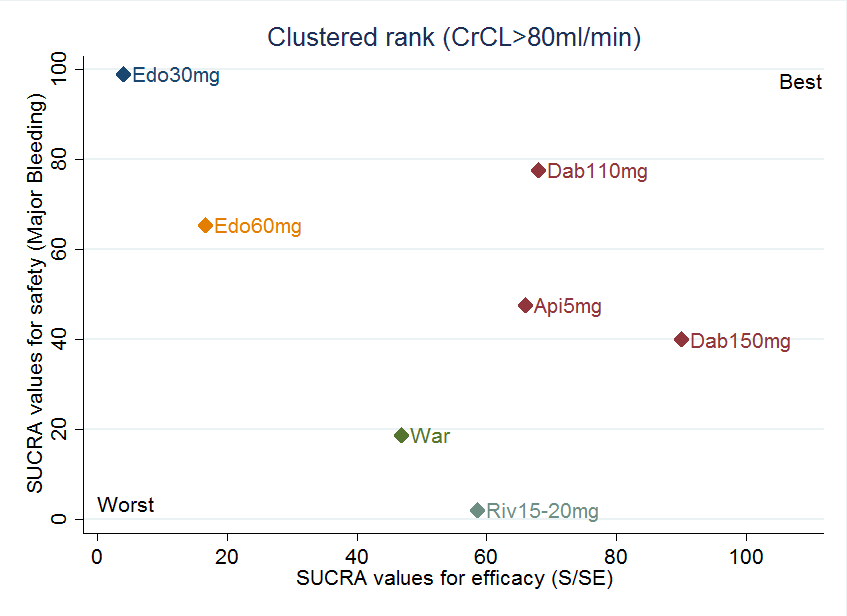


**Figure S6: Sensitivity Analysis of Forrest Plot for Efficacy in Patients with Moderate Renal Impairment**


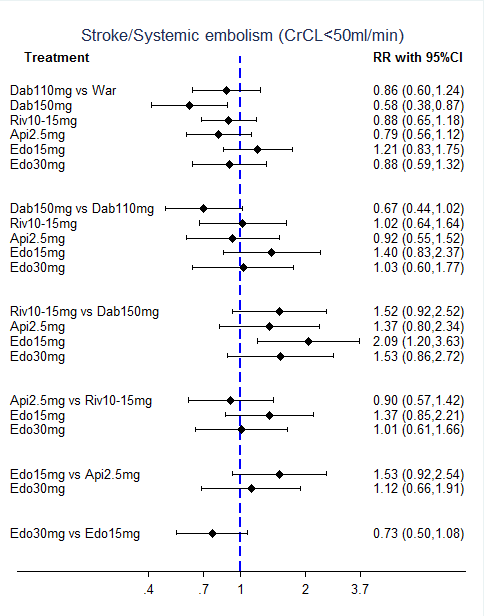


**Figure S7: Sensitivity Analysis of SUCRA Ranking for Efficacy in Patients with Moderate Renal Impairment**

**
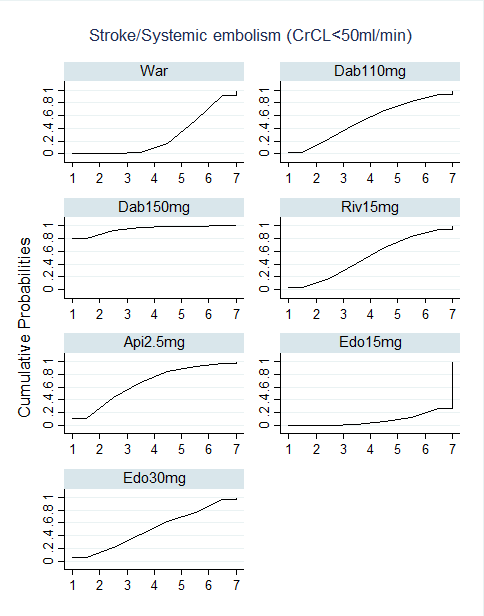
**

**Figure S8: Sensitivity Analysis of Forrest Plot for Safety in Patients with Moderate Renal Impairment**

**
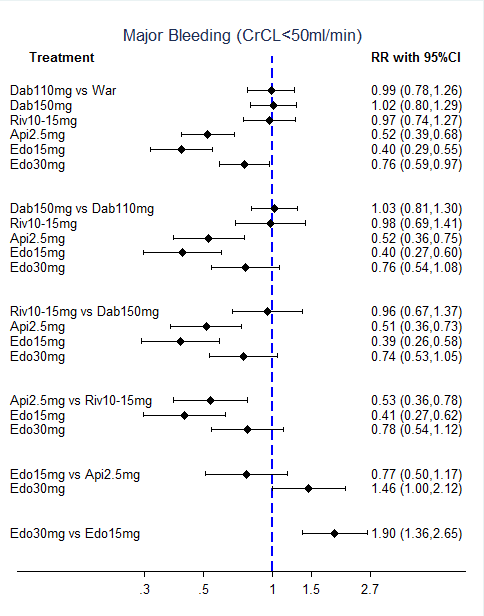
**

**Figure S9: Sensitivity Analysis of SUCRA Ranking for Safety in Patients with Moderate Renal Impairment**

**
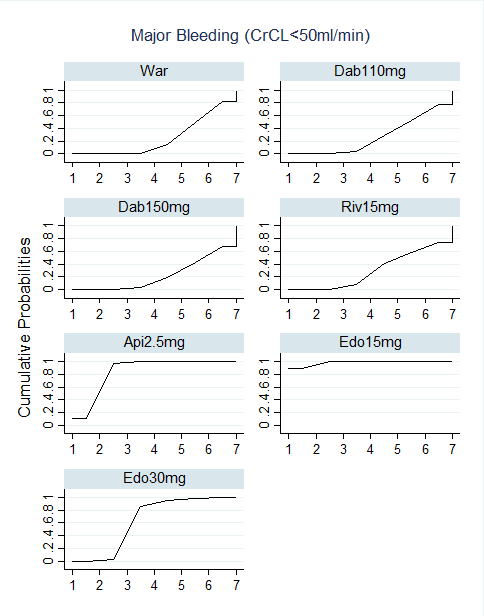
**

**Figure S10: Sensitivity Analysis of Clustered Ranking for Both Efficacy and Safety in Patients with Moderate Renal Impairment**

**
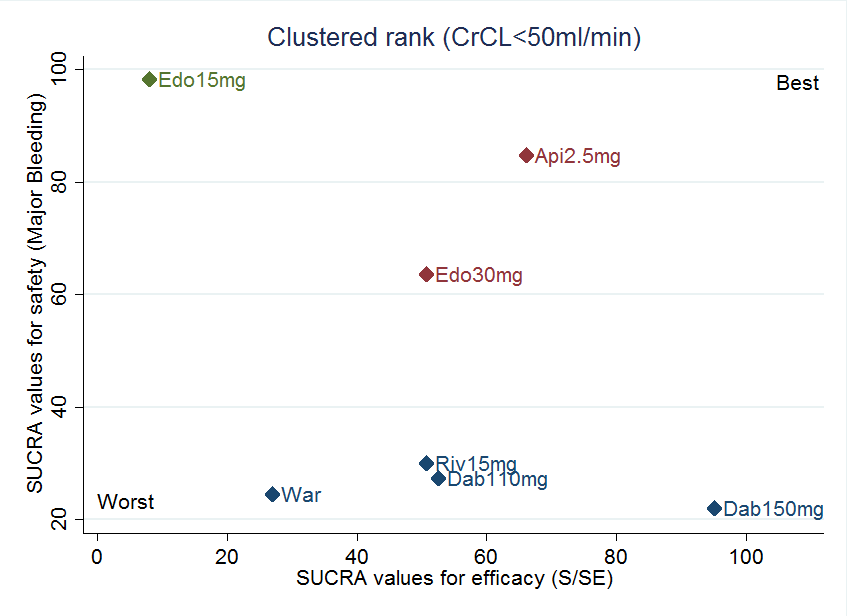
**

**Legends**

**Tables**

**Table S1. Quality Assessment Results of Included Randomized Controlled Trials**

**Figures**

**Figure S1.** **Sensitivity Analysis of Forrest Plot for Efficacy in Patients with Normal Renal Function**

War indicates Warfarin. Dab110mg indicates Dabigatran 110mg. Dab150mg indicates Dabigatran 150mg. Riv20mg indicates Rivaroxaban 20mg. Api5mg indicates Apixaban 5mg. Edo30mg indicates Edoxaban 30mg. Edo60mg indicates Edoxaban 60mg.

**Figure S2: Sensitivity Analysis of SUCRA Ranking for Efficacy in Patients with Normal Renal Function**

War indicates Warfarin. Dab110mg indicates Dabigatran 110mg. Dab150mg indicates Dabigatran 150mg. Riv20mg indicates Rivaroxaban 20mg. Api5mg indicates Apixaban 5mg. Edo30mg indicates Edoxaban 30mg. Edo60mg indicates Edoxaban 60mg.

**Figure S3: Sensitivity Analysis of Forrest plot for Safety in Patients with Normal Renal Function**

War indicates Warfarin. Dab110mg indicates Dabigatran 110mg. Dab150mg indicates Dabigatran 150mg. Riv20mg indicates Rivaroxaban 20mg. Api5mg indicates Apixaban 5mg. Edo30mg indicates Edoxaban 30mg. Edo60mg indicates Edoxaban 60mg.

**Figure S4: Sensitivity Analysis of SUCRA Ranking for Safety in Patients with Normal Renal Function**

War indicates Warfarin. Dab110mg indicates Dabigatran 110mg. Dab150mg indicates Dabigatran 150mg. Riv20mg indicates Rivaroxaban 20mg. Api5mg indicates Apixaban 5mg. Edo30mg indicates Edoxaban 30mg. Edo60mg indicates Edoxaban 60mg.

**Figure S5: Sensitivity Analysis of Clustered Ranking for Both Efficacy and Safety in Patients with Normal Renal Function**

SUCRA indicates the surface under the cumulative ranking curves. S/SE indicates stroke or systemic embolism. War indicates Warfarin. Dab110mg indicates Dabigatran 110mg. Dab150mg indicates Dabigatran 150mg. Riv20mg indicates Rivaroxaban 20mg. Api5mg indicates Apixaban 5mg. Edo30mg indicates Edoxaban 30mg. Edo60mg indicates Edoxaban 60mg.

**Figure S6. Sensitivity Analysis of Forrest Plot for Efficacy in Patients with Moderate Renal Impairment**

War indicates Warfarin. Dab110mg indicates Dabigatran 110mg. Dab150mg indicates Dabigatran 150mg. Riv15mg indicates Rivaroxaban 15mg. Api2.5mg indicates Apixaban 2.5mg. Edo15mg indicates Edoxaban 15mg. Edo30mg indicates Edoxaban 30mg.

**Figure S7. Sensitivity Analysis of SUCRA Ranking for Efficacy in Patients with Moderate Renal Impairment**

War indicates Warfarin. Dab110mg indicates Dabigatran 110mg. Dab150mg indicates Dabigatran 150mg. Riv15mg indicates Rivaroxaban 15mg. Api2.5mg indicates Apixaban 2.5mg. Edo15mg indicates Edoxaban 15mg. Edo30mg indicates Edoxaban 30mg.

**Figure S8. Sensitivity Analysis of Forrest Plot for Safety in Patients with Moderate Renal Impairment**

War indicates Warfarin. Dab110mg indicates Dabigatran 110mg. Dab150mg indicates Dabigatran 150mg. Riv15mg indicates Rivaroxaban 15mg. Api2.5mg indicates Apixaban 2.5mg. Edo15mg indicates Edoxaban 15mg. Edo30mg indicates Edoxaban 30mg.

**Figure S9: Sensitivity Analysis of SUCRA Ranking for Safety in Patients with Moderate Renal Impairment**

War indicates Warfarin. Dab110mg indicates Dabigatran 110mg. Dab150mg indicates Dabigatran 150mg. Riv15mg indicates Rivaroxaban 15mg. Api2.5mg indicates Apixaban 2.5mg. Edo15mg indicates Edoxaban 15mg. Edo30mg indicates Edoxaban 30mg.

**Figure S10. Sensitivity Analysis of Clustered Ranking for Both Efficacy and Safety in Patients with Moderate Renal Impairment**

SUCRA indicates the surface under the cumulative ranking curves. S/SE indicates stroke or systemic embolism. War indicates Warfarin. Dab110mg indicates Dabigatran 110mg. Dab150mg indicates Dabigatran 150mg. Riv15mg indicates Rivaroxaban 15mg. Api2.5mg indicates Apixaban 2.5mg. Edo15mg indicates Edoxaban 15mg. Edo30mg indicates Edoxaban 30mg.

**References**

1. S.J. [Connolly](https://www.ncbi.nlm.nih.gov/pubmed/?term=Connolly%20SJ%5BAuthor%5D&cauthor=true&cauthor_uid=19717844), M.D. [Ezekowitz](https://www.ncbi.nlm.nih.gov/pubmed/?term=Ezekowitz%20MD%5BAuthor%5D&cauthor=true&cauthor_uid=19717844), S. [Yusuf](https://www.ncbi.nlm.nih.gov/pubmed/?term=Yusuf%20S%5BAuthor%5D&cauthor=true&cauthor_uid=19717844), J. [Eikelboom](https://www.ncbi.nlm.nih.gov/pubmed/?term=Eikelboom%20J%5BAuthor%5D&cauthor=true&cauthor_uid=19717844), J. [Oldgren](https://www.ncbi.nlm.nih.gov/pubmed/?term=Oldgren%20J%5BAuthor%5D&cauthor=true&cauthor_uid=19717844),  A. [Parekh,](https://www.ncbi.nlm.nih.gov/pubmed/?term=Parekh%20A%5BAuthor%5D&cauthor=true&cauthor_uid=19717844) et al., Dabigatran versus warfarin in patients with atrial fibrillation, N. Engl. J. Med. 361 (12) (2009) 1139-1151.
2. C.B. [Granger](https://www.ncbi.nlm.nih.gov/pubmed/?term=Granger%20CB%5BAuthor%5D&cauthor=true&cauthor_uid=21870978), J.H. [Alexander](https://www.ncbi.nlm.nih.gov/pubmed/?term=Alexander%20JH%5BAuthor%5D&cauthor=true&cauthor_uid=21870978), J.J. [McMurray](https://www.ncbi.nlm.nih.gov/pubmed/?term=McMurray%20JJ%5BAuthor%5D&cauthor=true&cauthor_uid=21870978), R.D. [Lopes](https://www.ncbi.nlm.nih.gov/pubmed/?term=Lopes%20RD%5BAuthor%5D&cauthor=true&cauthor_uid=21870978), E.M. [Hylek](https://www.ncbi.nlm.nih.gov/pubmed/?term=Hylek%20EM%5BAuthor%5D&cauthor=true&cauthor_uid=21870978), M. [Hanna](https://www.ncbi.nlm.nih.gov/pubmed/?term=Hanna%20M%5BAuthor%5D&cauthor=true&cauthor_uid=21870978), et al., Apixaban versus Warfarin in Patients with Atrial Fibrillation, N. Engl. J. Med. 365 (11) (2011) 981-992.
3. 9. M. [Hori](https://www.ncbi.nlm.nih.gov/pubmed/?term=Hori%20M%5BAuthor%5D&cauthor=true&cauthor_uid=22664783), M. [Matsumoto](https://www.ncbi.nlm.nih.gov/pubmed/?term=Matsumoto%20M%5BAuthor%5D&cauthor=true&cauthor_uid=22664783), N. [Tanahashi](https://www.ncbi.nlm.nih.gov/pubmed/?term=Tanahashi%20N%5BAuthor%5D&cauthor=true&cauthor_uid=22664783), S. [Momomura](https://www.ncbi.nlm.nih.gov/pubmed/?term=Momomura%20S%5BAuthor%5D&cauthor=true&cauthor_uid=22664783), S. [Uchiyama](https://www.ncbi.nlm.nih.gov/pubmed/?term=Uchiyama%20S%5BAuthor%5D&cauthor=true&cauthor_uid=22664783), S. [Goto](https://www.ncbi.nlm.nih.gov/pubmed/?term=Goto%20S%5BAuthor%5D&cauthor=true&cauthor_uid=22664783), et al., Rivaroxaban vs. Warfarin in Japanese Patients With Atrial Fibrillation, Circ. J. 76 (9) (2012) 2104-2111.
4. 10. M.R. [Patel](https://www.ncbi.nlm.nih.gov/pubmed/?term=Patel%20MR%5BAuthor%5D&cauthor=true&cauthor_uid=21830957), K.W. [Mahaffey](https://www.ncbi.nlm.nih.gov/pubmed/?term=Mahaffey%20KW%5BAuthor%5D&cauthor=true&cauthor_uid=21830957), J. [Garg](https://www.ncbi.nlm.nih.gov/pubmed/?term=Garg%20J%5BAuthor%5D&cauthor=true&cauthor_uid=21830957), G. [Pan](https://www.ncbi.nlm.nih.gov/pubmed/?term=Pan%20G%5BAuthor%5D&cauthor=true&cauthor_uid=21830957), D.E. [Singer](https://www.ncbi.nlm.nih.gov/pubmed/?term=Singer%20DE%5BAuthor%5D&cauthor=true&cauthor_uid=21830957), W. [Hacke](https://www.ncbi.nlm.nih.gov/pubmed/?term=Hacke%20W%5BAuthor%5D&cauthor=true&cauthor_uid=21830957), et al., Rivaroxaban versus warfarin in nonvalvular atrial fibrillation, N. Engl. J. Med. 365 (10) (2011) 883-891.
5. 11. R.P. [Giugliano](https://www.ncbi.nlm.nih.gov/pubmed/?term=Giugliano%20RP%5BAuthor%5D&cauthor=true&cauthor_uid=24251359), C.T. [Ruff](https://www.ncbi.nlm.nih.gov/pubmed/?term=Ruff%20CT%5BAuthor%5D&cauthor=true&cauthor_uid=24251359), E. [Braunwald](https://www.ncbi.nlm.nih.gov/pubmed/?term=Braunwald%20E%5BAuthor%5D&cauthor=true&cauthor_uid=24251359), S.A. [Murphy](https://www.ncbi.nlm.nih.gov/pubmed/?term=Murphy%20SA%5BAuthor%5D&cauthor=true&cauthor_uid=24251359), S.D. [Wiviott](https://www.ncbi.nlm.nih.gov/pubmed/?term=Wiviott%20SD%5BAuthor%5D&cauthor=true&cauthor_uid=24251359), J.L. [Halperin](https://www.ncbi.nlm.nih.gov/pubmed/?term=Halperin%20JL%5BAuthor%5D&cauthor=true&cauthor_uid=24251359), et al., Edoxaban versus warfarin in patients with atrial fibrillation, N. Engl. J. Med. 369 (22) (2013) 2093-2104.
